# May Strenuous Endurance Sports Activity Damage the Cardiovascular System of Healthy Athletes? A Narrative Review

**DOI:** 10.3390/jcdd9100347

**Published:** 2022-10-10

**Authors:** Francesca Graziano, Vencel Juhasz, Giulia Brunetti, Alberto Cipriani, Liliana Szabo, Béla Merkely, Domenico Corrado, Flavio D’Ascenzi, Hajnalka Vago, Alessandro Zorzi

**Affiliations:** 1 Department of Cardiac, Thoracic and Vascular Sciences and Public Health, University of Padova, 35122 Padova, Italy; 2 Heart and Vascular Center of Semmelweis University, Hataror Rd. 18, 1122 Budapest, Hungary; 3 Department of Sports Medicine, Semmelweis University, Gaal Jozsef Str. 9-11, 1122 Budapest, Hungary; 4 Department of Medical Biotechnologies, Division of Cardiology, University of Siena, 53100 Siena, Italy

**Keywords:** athletes, atrial fibrillation, arrhythmias, cardiac magnetic resonance, electrocardiogram, ECG, exercise, long-QT, myocardial fibrosis, sports cardiology, troponin

## Abstract

The positive effects of physical activity are countless, not only on the cardiovascular system but on health in general. However, some studies suggest a U-shape relationship between exercise volume and effects on the cardiovascular system. On the basis of this perspective, moderate-dose exercise would be beneficial compared to a sedentary lifestyle, while very high-dose physical activity would paradoxically be detrimental. We reviewed the available evidence on the potential adverse effects of very intense, prolonged exercise on the cardiovascular system, both acute and chronic, in healthy athletes without pre-existing cardiovascular conditions. We found that endurance sports activities may cause reversible electrocardiographic changes, ventricular dysfunction, and troponin elevation with complete recovery within a few days. The theory that repeated bouts of acute stress on the heart may lead to chronic myocardial damage remains to be demonstrated. However, male veteran athletes with a long sports career show an increased prevalence of cardiovascular abnormalities such as electrical conduction delay, atrial fibrillation, myocardial fibrosis, and coronary calcifications compared to non-athletes. It must be underlined that the cause–effect relationship between such abnormalities and the exercise and, most importantly, the prognostic relevance of such findings remains to be established.

## 1. Introduction

The positive effects of physical activity, not just on the cardiovascular system but on health in general, are countless [1,2,3]. However, intense physical exercise may trigger malignant ventricular arrhythmias and promote disease progression in patients with underlying heart disease [4]. Whether strenuous and prolonged sports practice may carry short- and long-term adverse consequences to the heart is a matter of debate. According to one theory, a U-shaped relationship exists between dose of physical activity (pace, quantity, and frequency) and cardiovascular system effects. On the basis of this perspective, moderate-dose exercise would be beneficial compared to a sedentary lifestyle, but very high dose of physical activity would paradoxically be detrimental [5].

This review discusses the available evidence on the potential adverse effects of intense, prolonged endurance exercise on the cardiovascular system, both acute and chronic, in healthy athletes. The effects and risks of sports practice in individuals with pre-existing cardiovascular conditions are not addressed.

## 2. Acute Effects

Prolonged strenuous exercise has profound acute effects on both structure and function of the heart. The right side of the heart is more sensitive to volume and pressure overload induced by exercise than the left side due to its thinner wall and lower resting pressure of the right chambers [6]. During exercise, the progressive increase in pulmonary vascular resistance leads to higher relative wall stress in the right ventricle (RV) when compared to the left ventricle (LV) [7]. This can cause an acute overload of the right heart that can lead to acute RV dysfunction, the occurrence of arrhythmias, and electrocardiographic (ECG) abnormalities.

### 2.1. Acute ECG Abnormalities

Lord et al. [8] described 12-lead ECG findings following a 100-mile ultra-marathon and demonstrated acute changes reflecting RV overload. Specifically, they described an increase in the summated R wave in V1 and S wave in V5 (i.e., the Sokolow–Lyon criteria for RV hypertrophy) and in the elevation of the J point as well as in the prevalence of incomplete right bundle branch block and T-wave inversion. The echocardiographic assessment confirmed the RV overload following the race. The same authors showed that also the right precordial leads (V1R-V6R) show acute modifications after an ultramarathon [9].

**Figure 1:**
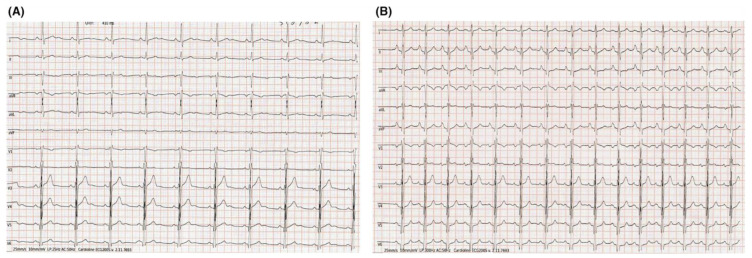
Acute ECG changes reflecting right heart overload. ECG of a top-level competitive athlete the day before (**A**) and immediately after (**B**) running a 50 km ultramarathon. After the race, the ECG showed higher P-wave voltages and a rightward shift in the QRS axis. Adapted from D’Ascenzi et al. [10].

D’Ascenzi et al. [10] evaluated the acute ECG modifications following endurance exercise (50 km ultramarathon). They found that ECG markers of right heart overload developed in a sizeable proportion of athletes, with a risen voltage of P wave, right atrial (RA) enlargement, increase in R-wave voltage in V1, and a rightward shift in the QRS axis as compared with pre-race data, which were more evident in athletes with the best performance during the race (Figure 1).

In a group of 10 athletes running a mountain marathon, a post-race prolongation of the signal-averaged P-wave duration was observed [11]. This ECG sign was accompanied by the elevation of several inflammatory markers, as well as atrial natriuretic peptide and high-sensitivity troponin, and the authors hypothesized that atrial inflammation might underline the prolongation of the intra-atrial conduction times. 

In summary, the studies agree that following an ultramarathon run, the ECG shows modifications reflecting the right heart overload.

### 2.2. Acute Atrial Dysfunction

Atrial dilatation is typical of the athlete’s heart, but the atrial function is usually normal [12,13,14,15]. However, acute strenuous exercise may acutely impair atrial function. The high degree of stress on the myocardial structure during endurance sports has an impact, especially on the right heart cavities; however, some evidence suggests that the left atrium (LA) may also be affected. 

After a marathon, a reduction in LA diastolic and systolic peak deformation was demonstrated through myocardial speckle-tracking echocardiography in 17 healthy adult men [16]. The mechanism seemed to be secondary to impairment of the LV diastolic relaxation. Sanz-de la Garza et al. [17] analyzed the acute effects of a trail race of three lengths (14, 35, and 56 Km). They found that the RA reservoir and contractile function decreased after the longest race. The medium distance impacted the RA reservoir but not the contractile function, while athletes who had run the shorter distance did not exhibit modifications in the RA function. A similar trend was observed for the LA that was affected to a lesser degree than the RA. The authors also found high interindividual variability in atrial response to exercise among athletes running the same distance. Chen et al. [18] evaluated atrial function by feature-tracking cardiac magnetic resonance (CMR) after a triathlon completion: they found that LA global longitudinal strain, but not RA, decreased after exercise.

Different results were obtained by Cavigli et al. [19], who studied the echocardiographic and electrocardiographic changes of a group of 68 master athletes (≥40 years old) participating in a 50 km ultra-marathon. They found that mean biatrial size and function were within normal values and did not differ after the race compared with pre-race values. These data disproved the hypothesis of an acute atrial dysfunction induced by ultra-endurance exercise, although reporting of mean values only did not allow assessing whether post-race differences were observed, at least in a subgroup of athletes.

In summary, discrepancies exist among different studies concerning the acute effects of intense exercise on atrial function. Even if prolonged exercise can cause acute atrial dysfunction, preliminary evidence suggests that this is a transient and reversible effect [20].

### 2.3. Acute Ventricular Dysfunction

Exercise causes an increase in cardiac output due to higher heart rate and cardiac contractility. A noted hypothesis, however, presumes that maintaining a high cardiac workload for a prolonged time may result in a transient cardiac dysfunction, i.e., a form of exercise-induced “cardiac fatigue” [21]. Saltin and Stenberg described this phenomenon for the first time in 1964 by reporting that the stroke volume decreased after 3 h of exercise in four athletes [22]. The short-term effect of prolonged endurance exercise on LV function was analyzed in a meta-analysis of 23 studies published in 2002: reduction in LV ejection fraction with exercise was reported more frequently in untrained subjects performing moderate duration physical activity (>3h) and in trained athletes performing ultra-endurance races (>10.5 h). Recovery of LV ejection fraction to previous values is typically described after 48 h [23]. In 2015, a meta-analysis that included studies using advanced imaging techniques found that prolonged exercise reduces LV global longitudinal strain and twisting [24]. Alexoius et al. studied the acute effects of exhaustive 25 km open-sea swimming on LV function and morphology in a group of 20 elite male swimmers (22.3 ± 4.1 years). The result was that prolonged exhaustive swimming was associated with depressed LV function, as suggested by reduced stroke volume, ejection fraction, and LV fractional shortening. According to the authors, increased afterload would justify this finding [25]. In contrast, a third meta-analysis also published in 2015 [26] found that prolonged endurance exercise does not have an impact on LV function. LV ejection fraction, strain, strain rates, rotation, rotation rates, and torsion were also unaltered in a cohort of non-elite male runners after the London marathon [27], and in a cohort of master amateur athletes running a 50 km ultramarathon [28]. Gajda et al. demonstrated that prolonged intense swimming did not affect biventricular function in 14 swimmers (13–67 years) participating in an ultramarathon, swimming for 500 km (in 5 km intervals) [29].

As previously discussed, the RV function may be more profoundly affected than the LV by cardiac fatigue, secondary to an increase in volume and pulmonary systolic pressure [30,31]. A meta-analysis of 14 studies examining RV function following an event of at least 90 min duration found that parameters of RV function tend to worsen after exercise with no significant difference according to exercise time > or <6 h [26]. In contrast, in 35 runners participating in the London marathon (aged 18–50 years), no significant changes were found after the race in RV function [16]. In the same way, among 68 master athletes participating in a 50 km ultramarathon, mean RV dimensions and functions did not change after the race. In only four athletes, a reduction of RV strain, not accompanied by other signs of dysfunction, was observed [28]. 

In summary, discrepancies exist in the literature about the acute effects of prolonged exercise on ventricular function. These differences may be linked to different methods, study samples, and exercise duration. It is likely that only intense prolonged exercise may lead to transient ventricular dysfunction in a subset of athletes. All studies agree that ventricular “fatigue” is a transient phenomenon with complete recovery of ventricular function [7]. The theory that repeated bouts of transient myocardial dysfunction may eventually lead to permanent damage (so-called “exercise-induced cardiomyopathy”) is discussed below.

### 2.4. Atrial and Ventricular Ectopic Beats

At present, there is no evidence that athletes show an increase in the ectopic atrial burden compared to their sedentary counterparts [32] or that endurance exercise acutely triggers atrial ectopic beats [19,33]. These recently published findings are relevant because an increased atrial ectopic activity is one of the potential mechanisms of the increased prevalence of atrial fibrillation (AF) in athletes (see below).

The ventricular ectopic burden does not seem to differ between athletes and non-athletes [34,35,36,37], with only one study reporting a higher prevalence of complex ventricular arrhythmias at 24 h Holter monitoring ECG in 40 young endurance athletes than in 40 sedentary individuals [38]. Recent investigations compared the prevalence and determinants of ventricular ectopic beats on 24 h Holter monitoring among young [39] and middle-aged endurance athletes [40], finding no differences compared with non-athletes. Most athletes showed no or very few premature ventricular beats during the 24 h recording. 

**Figure 2:**
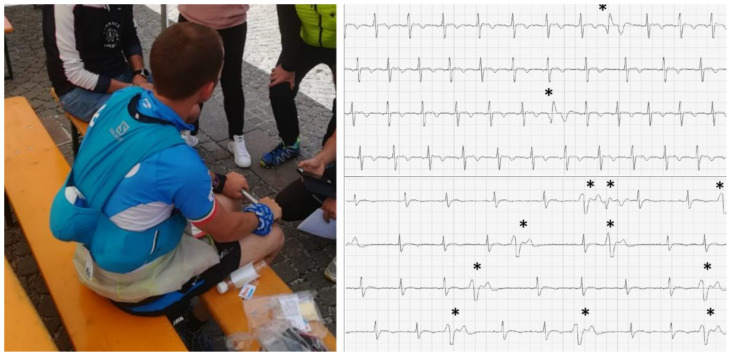
Example of two post-run ECG recordings with a portable single-lead electrocardiogram device showing premature ventricular beats (*) that were not present before the race. Reproduced with permission from Zorzi et al. [39].

Zorzi et al. [41] described the ECG changes of a group of athletes after an endurance (48 km) and ultra-endurance (120 km) high-altitude race. They used a portable single-lead ECG device and recorded a 1 min tracking before and immediately after the run. After the race, the athletes showed a higher heart rate, a similar QRS duration, and a longer QTc interval duration. The number of athletes showing at least one premature ventricular beat increased significantly after the race from 0.5% to 3.3%. However, most participants did not show any arrhythmia after strenuous exercise (Figure 2).

Cavigli et al. recorded the heart rhythm during a 50 km ultramarathon and found that 24% showed PVBs, but in only a minority of them, the PVBs were distinctively exercise induced (i.e., 7%). In agreement, other studies have reported no significant effect of a marathon race on ventricular arrhythmias in male endurance athletes [42,43]. Hence, current evidence does not support the perspective that sports activity increases the burden of atrial or ventricular arrhythmias in healthy individuals. Similarly, no increase in the incidence of atrial or ventricular ectopic beats is observed in most athletes, even during and after a prolonged period of exercise.

### 2.5. Troponin Elevation

Cardiac troponin (cTn) elevation is a frequently investigated factor associated with potential myocardial injury in athletes. Minor elevation in serum cTn levels after intensive training is a widely observed phenomenon [44]. According to a meta-analysis published in 2007 [45], post-exercise cTn levels exceeded the assay detection limit in 47% of the participating athletes, where running events may be affected more than cycling competitions. Several factors may predict the amount of cTn measured post-exercise, of which training intensity, training duration, age, and blood pressure seem to be the most relevant parameters [46,47,48,49]. Peak post-exercise cTn concentrations are usually 1–3 times higher than the upper reference limit returning to baseline after 48 to 72 h post-exercise [50]. In marathon runners, elevation in cTn may reach up to 10 times the baseline value [51].

The clinical relevance of exercise-related cTn elevation is disputed. Traditionally, exercise-induced cTn elevation has been regarded as a benign phenomenon. Although the exact mechanism remains to be determined, it does not necessarily mark definite necrosis of cardiac muscle cells but may also result from reversible injury attributable to cell wounds, cytoplasmatic blebbing, extracellular vesicle release, or extracardiac causes [50]. 

Some studies suggest that cTn release after sports activity may be associated with post-exercise ventricular dysfunction, but other investigations did not confirm this, and the strength of the association was moderate [50].

Whether cTn after exercise may be a marker of subclinical coronary artery disease (CAD) has also been investigated. Two studies correlated the association between calcium score on coronary computed tomography and post-exercise cTn release but found no association [52,53]. The NEEDED 2014 study assessed cTn levels in 1002 cyclists after a 91 km mountain bike race. The authors performed coronary computed tomography in the 80 cyclists with the highest post-exercise cTn (196 to 7919 ng/L) and 40 control cyclists with post-exercise cTn levels in line with the overall study sample (7–189 ng/L). They found obstructive CAD in 8/80 (10%) of the “highest cTn cohort” versus 1/40 (2.5%) of the “control cohort” [54]. The research team followed up with the participants 5 years after the race. Of the 1002 subjects, only 1.2% suffered a cardiovascular event. The authors did not find an association between cTn levels above the 99th percentile and cardiovascular event rate [55]. Similarly, Möhlenkamp et al. found no association between post-exercise cTn concentrations and coronary events during follow-up in German marathon runners. 

Taken together, the results of these studies suggest that in most athletes, mild cTn elevation after intense exercise is benign and not associated with an adverse prognosis, although particularly high values may underline concealed CAD [53]. Of note, the same consideration of the benign nature of post-exercise cTn elevation may not apply to less vigorous physical activities. A study on 725 physically active subjects, relatively older (mean age of 61 years), was assessed after a long-distance recreational walking event. Blood sampling took place immediately after finishing. Cardiac troponin I levels independently predicted higher mortality and cardiovascular events with an adjusted hazard ratio of 2.48 [50].

Finally, it may be postulated that repeated bouts of myocyte death with cTn release following strenuous exercise may result in non-ischemic myocardial scarring. Möhlenkamp et al. examined the possible associations of hsTnI elevation and CMR findings in 74 German marathon runners, of whom 9 showed late gadolinium enhancement (LGE) [53]. Athletes with LGE had higher troponin elevation than those without; however, the type and distribution of LGE were not specified, and we now know that certain LGE patterns (such as the “junctional pattern”) are non-pathological [56]. Tahir et al. found a high prevalence of LGE among triathletes, but those with and without LGE had comparable cTn concentrations after exercise [57]. Other CMR studies assessing myocardial oedema and LGE found no pathological alterations in marathon participants with increased cTn levels [58,59,60]. While CMR may not be sensitive enough to visualize small myocardial necroses after one bout of prolonged endurance training, there appears to be no demonstrated association between post-exercise cTn and myocardial fibrosis, as suggested by LGE.

In summary, the postexercise release of cTn rarely underlies a subclinical cardiac disease and can be considered a physiological phenomenon observed in athletes of all ages. Future studies are needed to establish the influence of confounders (such as age, sex, sport type, intensity and duration) and individualized ranges of normality of post-exercise cTn levels in athletes [61].

## 3. Chronic Effects

### 3.1. Atrioventricular Conduction Defects

Bradycardia is typically observed in trained athletes and is interpreted as a normal finding. It may persist 10 years after the end of the athletic career [62,63,64]. In the same way, first-degree atrioventricular block and second-degree type 1 atrioventricular block are considered normal in highly trained athletes in the absence of symptoms and with regress during effort [65]. However, some data suggest that former and veteran athletes show a significantly higher frequency of conduction disorder and hemodynamically significant asystolic pauses that require pacemaker implantation in comparison with age-matched controls [66,67,68]. Usually, these electrical alterations in athletes are considered due to increased vagal tone [69]. However, in some cases, they persist even under a complete pharmacological block of the autonomic nervous system [70]. More recently, animal models demonstrated an electrophysiological remodeling of the sinus and atrioventricular nodes due to an exercise-induced reduction in density of some ion channels (I_Ca,L_ e I_f_) controlled by a network of microRNA regulating channels transcription [71]. Even if the evidence is still preliminary and these findings must be replicated in humans, they can be considered as a possible explanation for node dysfunction in some former athletes.

### 3.2. QT-Interval Prolongation

Some abnormalities in the ventricular repolarization are considered normal in competitive athletes, including some degree of QTc prolongation that is modest and not directly related to long QT syndrome (LQTS) [72]. Indeed, the cut-off values to define prolonged QTc interval in athletes are higher than those recommended for the general population [65]. 

**Figure 3:**
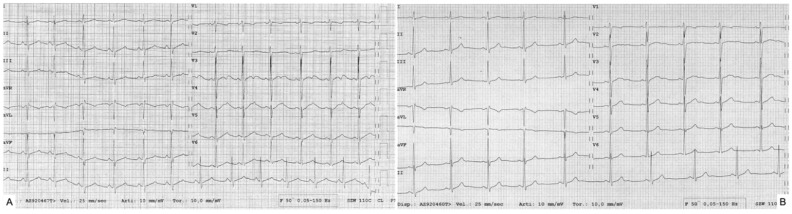
**A case of sport-induced long-QT syndrome.** Male, 15-year-old, plays water polo at a competitive level (training >10 h/week). Negative medical and family history. ECG at pre-participation screening: QTc 597 ms, HR 79 bpm (**A**), with notched morphology of T waves. Detraining was recommended. Genetic testing of the genes associated with LQTS resulted negative. ECG after 3 months of detraining: QTC 394 ms, HR 58 bpm (**B**).

However, a new condition has been recently described, the so-called “sport-related LQTS”. It refers to the observation that some highly trained athletes develop marked QTc prolongation with T-wave alterations, such as notched and biphasic T-wave morphologies, overlapping with patients affected by genetically determined long-QT syndrome [73]. Most importantly, these alterations are reversible with detraining, and genetic testing is negative (Figure 3). 

Dagradi et al. [74] studied 310 athletes referred to their center because of QTc interval prolongation detected during pre-participation screening. Most of them received a diagnosis of LQTS because of positive genetic testing and/or unequivocal ECG abnormalities, even in the absence of genetic alteration. Instead, 33 athletes were asymptomatic, with no family history and negative genetic testing and presented a complete ECG normalization after detraining. Moreover, some of them presented the same ECG abnormalities after retraining. This condition can be considered a form of acquired LQTS, even if the underlying mechanism is still not known. 

The hypothesis is that predisposed athletes (possibly because of a still unknown genetic background) react to the increased stretch of myocardial cells due to exercise by increasing the intracellular release of Ca^2+^. Another theory is that intense sport could lead to the downregulation of repolarizing potassium channels. The result is a prolongation of the action potential with increased calcium recruitment, which is a positive effect from a mechanical point of view because it enhances myocardial contractility, but can increase the arrhythmic risk [75]. While it is known that drug-induced LQTS is correlated with life-threatening arrhythmias [76], sport-induced LQTS seems to be a more benign condition. Further studies are needed to better understand this abnormality, stratify the risk, and to define if any therapy is needed, especially for patients who want to restart physical activity.

### 3.3. Atrial Fibrillation

Regular physical activity helps prevent AF by modifying numerous risk factors. In a review by Elliott et al., an exercise volume of 210 min per week was found to reduce the risk of AF [77]. A good cardiorespiratory fitness level is also a protective factor against AF: those who perform more than 8 METs (or >28 mL/kg/min VO2) on CPET bear decreased risk. According to the assessment of 5962 veterans (mean age 56.8 years), exercise capacity is inversely related to AF incidence. Faselis et al. reported a 21% decrease in the AF risk for each 1 MET increase in exercise capacity [78]. Another study involving 5446 older adults (above 65 years old) assessed AF incidence depending on leisure time activity and exercise intensity. During a follow-up period of 12 years, 1061 new cases of AF were diagnosed in 47,280 person-years. Exercise intensity showed a U-shaped relationship with AF occurrence: light-to-moderate intensity was deemed a protective factor, whereas high-intensity physical activity did not show this tendency [79]. Naturally, this latter population seldom overlaps with elite athletes.

In contrast to physically active and fit individuals, AF is more prevalent in former male master athletes and high-endurance athletes, while women do not show this tendency. In a cohort of 52,755 cross-country skiers (high-intensity endurance discipline), the incidence of any arrhythmia (AF was the most common) increased with higher age and the number of completed races [80]. This finding is supported by numerous other studies where long-term endurance athletes such as marathon or ultramarathon runners and professional cyclists showed an up to a fivefold increase in AF prevalence [81,82,83]. A prospective examination called the third Tromso study included 20,484 adults whose physical activity and resting heart rate were measured [84]. The investigators found a 19% decrease in the risk of developing AF in the moderately active group, defined as individuals engaging in walking, cycling, or other forms of exercise at least 4 h per week. In a sex- and age-adjusted model, those performing low (i.e., sedentary lifestyle) versus high activity (recreational sports, heavy gardening at least 4 h per week) showed similar hazard ratios. Subjects with vigorous exercise regimes (hard training or competitors) bore higher but statistically insignificant hazard ratios for AF.

The pathophysiological hypothesis linking endurance exercise to AF risk in male master athletes is that the increased vagal tone in athletes decreases the atrial refractory period, which could facilitate re-entry and predispose to AF, especially because it is associated with an intermittent-exercise-related increase in sympathetic tone [84,85,86]. Indeed, AF in athletes typically occur at rest and especially during sleep when the vagal tone is higher. Moreover, animal studies demonstrated an association between atrial enlargement, progressive atrial fibrosis, and inflammation due to intense exercise and their correlation with the risk of AF [87]. The confirmation in humans of a structural atrial disease caused by exercise in intensively trained individuals is still pending. Of note, it seems that women are at a generally lower risk of developing AF than men because resting heart rate is usually higher, differences in vagal tone, smaller atria, and shorter P-wave duration, but these hypotheses are speculative and have not been demonstrated yet [88,89].

In summary, the available data suggest a U-shaped relationship between exercise dose and AF risk. According to the latest European Society of Cardiology guidelines, counselling is advised regarding the possible effects of long-lasting, high-intensity sports activity on AF risk, especially in middle-aged men [90].

### 3.4. Exercise-Induced Arrhythmogenic Right Ventricular Cardiomyopathy

**Figure 4:**
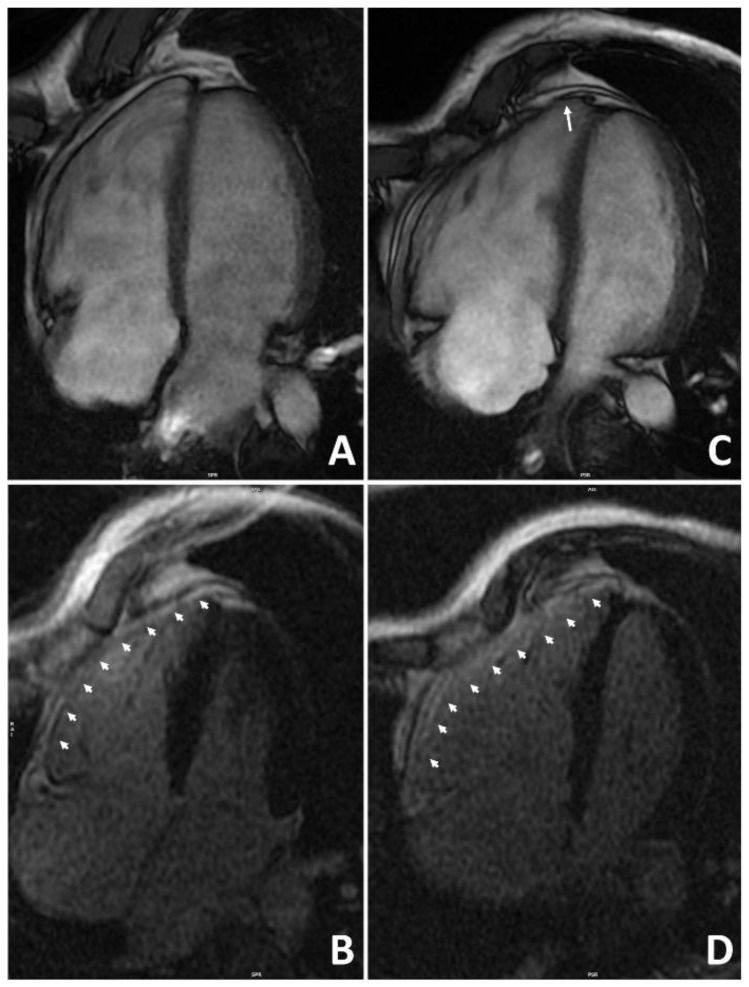
**Representative example of the role of endurance exercise in worsening the arrhythmogenic cardiomyopathy phenotype.** A 38-year-old runner received a diagnosis of arrhythmogenic cardiomyopathy after investigating ventricular arrhythmias and ECG abnormalities at preparticipation screening. Genetic testing was positive for plakophilin-2 gene mutation. At the time of diagnosis, cardiac magnetic resonance 4-chamber view on cine sequences found dilatation of the right ventricle with mild dysfunction (**A**) and diffuse right ventricular late-enhancement on post-contrast sequences (**B**). Although he was considered not eligible for competitive sports activity according to Italian law, he continued to practice high-intensity endurance training. After 4 years, repeat cardiac magnetic resonance showed a more enlarged right ventricle, an apical aneurysm (arrow), and a moderate right ventricular dysfunction (**C**). Post-contrast sequences confirmed diffuse right ventricular late enhancement (**D**). Reproduced with permission from Zorzi et al. [92].

Arrhythmogenic right ventricular cardiomyopathy (ARVC) is an inherited disease characterized by fibro-fatty replacement predominantly involving the RV (although some LV involvement is present in most cases). Clinically, it is characterized by ventricular arrhythmias and regional or global ventricular wall motion abnormalities [91]. It is undisputed that intense exercise plays an adverse effect on patients with ARVC by accelerating the disease process and triggering life-threatening arrhythmias (Figure 4) [92].

However, it has also been proposed that intense and prolonged endurance exercise may itself cause ARVC, so-called “exercise-induced arrhythmogenic cardiomyopathy”.

Exercise-induced arrhythmogenic cardiomyopathy as a separate entity was introduced by Heidbuchel et al. in 2003 [93]. They conducted a complex electrophysiological assessment of 46 high-level endurance athletes (80% cyclists and 17% long-distance runners) with probable ventricular arrhythmias. Only one athlete had a familial history suggestive of inherited ARVC. Fifty-nine percent of athletes fulfilled the contemporary Task Force Criteria for ARVC, and an additional thirty percent were classified as potential ARVC patients. Inducible arrhythmias during an electrophysiological study were predominantly (90%) left bundle branch morphology, suggestive of RV or septal origin. Despite the small number of subjects in this study, the authors proposed causality between endurance sports and the ARVC phenotype. La Gerche et al. found a transient deterioration of RV function following endurance exercise [94]. The authors hypothesized that, in some subjects, repeated bouts of RV damage following intense exercise may not heal completely but result in exercise-induced arrhythmogenic cardiomyopathy. Following these landmark studies, several other publications investigated exercise-induced arrhythmogenic cardiomyopathy [95].

Subsequent genotype–phenotype studies showed that ARVC features in subjects who engaged in high-intensity exercise were typically not associated with a gene mutation [96,97]. In the “John Hopkins registry” of ARVC, patients with negative genetic testing, particularly those under 25 years old, reported performing more intense exercise significantly before their diagnosis compared to those with positive genetic testing for desmosomal-gene mutation [97]. These observations further support the hypothesis that ARVC may be acquired through intense exercise. However, ARVC is very rare, and many studies demonstrated a lack of long-term adverse RV remodeling in large series of top-level athletes [98]. For this reason, it is plausible that a combination of a still unknown genetic predisposition and prolonged endurance exercise is needed to develop the disease.

### 3.5. Myocarditis and Left Ventricular Fibrosis

A non-ischemic myocardial scar is a CMR terminology that refers to the accumulation of gadolinium (i.e., LGE) in the ventricle’s subepicardial or mid-myocardial layers [98]. The gadolinium accumulates where the extracellular matrix is increased compared to the healthy myocardium, and for this reason, it is considered a marker of myocardial scar, which is indeed an accumulation of collagen in the extracellular matrix following a myocardial injury, cardiac stress, or infiltration of substances [99,100]. Although non-ischemic scars are found in various diseases (such as ARVC, hypertrophic cardiomyopathy, or dilated cardiomyopathy), they may also be found in isolation. An isolated non-ischemic LV scar is an emerging cause of life-threatening ventricular arrhythmias and sudden cardiac death in athletes [56], and some studies suggest that the prevalence of non-ischemic LGE (excluding the non-pathological junctional pattern) in athletes is higher than sedentary individuals. Besides peculiar gene mutations (e.g., Filamin C, Lamin A/C, desmoplakin) that can cause LV scarring, two main theories may explain the finding of a non-ischemic scar in an athlete: healed myocarditis [101] and exercise-induced myocardial damage with replacement fibrosis.

Elite athletes are at higher risk of myocarditis than the general population. Indeed, exercise has variable effects on the immunological system that depends on the intensity and duration of physical activity. While moderate exercise may improve immunological defense [102,103], intense and prolonged training can impair immunity; alter the T-cell response; and reduce the level of salivary secretory immunoglobulin A, lysozyme, and lactoferrin. These mechanisms increase the susceptibility of athletes to infections, especially of the upper respiratory tract, both after a single workout [104] and during chronic training [105,106,107,108]. Moreover, animal models have demonstrated that intense physical activity can worsen the pathobiological course of both viral and immune acute myocarditis [109,110]. A link between type/intensity of sports discipline and complicated myocarditis was described by Bouchau et al. [111], who identified a significant association between high static component sport and complicated myocarditis. Instead, athletes practicing endurance activities more frequently presented uncomplicated disease. The authors considered the endurance-exercise-induced downregulation of T-cell response as a possible explanation for reduced inflammation and necrosis, which represents the process of myocarditis. Conversely, power sport is characterized by upregulation of cellular immunity and cytotoxic damage [112].

The second theory linking sport to the non-ischemic scar is that intense exercise can cause cardiac damage and replacement fibrosis. In elite middle-aged and veteran athletes, biochemical evidence of abnormal collagen turnover was proven, with a higher level of plasma markers of collagen syntheses and degradation compared with age-matched sedentary controls [113]. Animal models have demonstrated that high-intensity endurance exercise can lead to myocardial fibrosis, and the cessation of training was able to arrest and reverse this pathological process [87], but these findings have not been confirmed in humans.

Table 1 summarizes the main studies reporting the prevalence and characteristics of late gadolinium enhancement in endurance athletes. Overall, the presence of non-ischemic LGE (i.e., excluding the subendocardial ischemic pattern) was demonstrated in a minority of endurance athletes. However, the subgroup of studies that included a control population showed a higher prevalence of LGE in athletes than in sedentary controls. In interpreting the results of the different investigations, it must be underlined that some authors considered the so-called junctional spotty pattern located at the attachment of RV wall to the septum as a sign of non-ischemic fibrosis. It is now known that the junctional LGE is a non-pathological finding in athletes, probably caused by the expansion of the interstitial space (rather than fibrosis) due to the constant flexing at this “hinge” point produced by both exercise and the right ventricular enlargement that attends long-term exercise training [56]. While the junctional pattern can be considered as a feature of the athlete’s heart, evidence on the cause–effect relationship between sports activity and other non-ischemic LGE patterns (particularly the infero-lateral LV stria) is still inconclusive.

**Figure 5:**
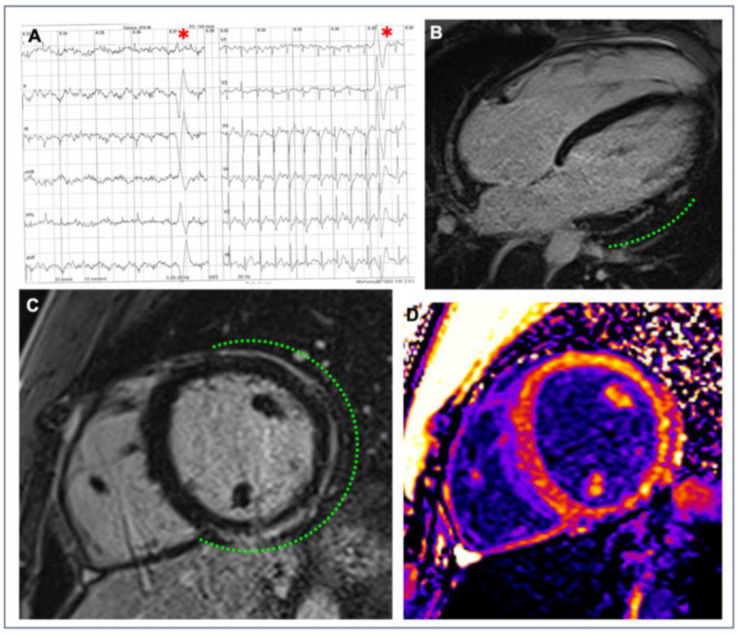
**Example of a myocardial scar in an athlete.** A competitive hockey player aged 26 at the pre-participation screening during exercise testing showed frequent PVBs with right bundle branch block/superior axis morphology at high workload ((**A**), red asterisks). Post-contrast sequences on CMR revealed a subepicardial stria of LGE involving the anterior, lateral, and inferior LV walls in their basal and medium portions, with a “ring-like” pattern (green dotted line; (**B**) 4-chamber view; (**C**) short-axis view). Increased signal in the correspondent areas of fibrosis in the native T1 mapping short-axis sequence (**D**). Reproduced with permission from Brunetti et al. [129].

More recent techniques, such as T1, T2 mapping, and extracellular volume (ECV), are used to detect diffuse myocardial fibrosis that cannot be assessed by conventional LGE technique. However, evidence in athletes is still inconsistent. A Scottish study observed no difference in native T1, T2 relaxation time, and ECV between athletes and controls [114]. In contrast, in a Turkish study, significantly higher native T1 values of the LV and interventricular septum were found in athletes compared with controls [115]. T1 mapping, ECV, and LGE were analyzed in a group of 78 male and female triathletes compared to a group of controls. Non-ischemic LGE was found in seven male triathletes, and it was correlated with higher systolic pressure at peak exercise, longer distances in swimming, and higher ECV values, indicating diffuse fibrosis [116]. Conversely, ECV and native T1 values were lower in athletes without LGE. It has to be underlined that the significance of an isolated increase in mapping values in athletes is still under evaluation, and it does not necessarily have a pathological significance [117] (Figure 5).

**Table 1:** Summary of studies investigating the prevalence and characteristics of late gadolinium enhancement in athletes. Yrs = years; LGE = late gadolinium enhancement.

Reference	Year	N° Cases	Mean Age	Males %	Controls	Inclusion Criteria	% LGE	LGE Patterns in Cases
Mousavi et al. [61]	2009	14	33	57	NO	Marathon runners, moderately trained	0	
Breuckmann et al. [118]	2009	102	57	100	YES	Marathon runners (≥5 marathons in the last 3 years), age ≥ 50 yrs old	12 (4 controls)	5: subendocardial 7: midmyocardial/subepicardial
O’Hanlon et al. [119]	2010	17	34	100	NO	Marathon runners mean 7 h training/week	0	
Oomanh et al. [120]	2011	15	32	47	NO	Half marathon runners, non-elite	0	
Wilson et al. [121]	2011	12	57	100	YES	Endurance elite, various sports, >50 yrs old	50 (0 controls)	4: junctional 1: subendocardial 1: subepicardial stria
Karlstedt et al. [122]	2012	25	55	84	NO	Elite athletes (≥3 marathons in the last 2 years), age > 50 yrs	9	2: subendocardial
La Gerche et al. [123]	2012	40	37	90	NO	Endurance, >10 h/training, high performance	13	1: junctional 4: spots in the septum
Erz et al. [124]	2013	45	35	100	NO	Endurance, >7 h/week for >2 yrs	2	1: inferior wal spot 1: lateral wall spot
Mangold et al. [125]	2013	95	33	77	NO	Elite athletes, various sports, training history ≥2 yrs and 15 h/week	2	2: spot-shaped pattern
Franzen et al. [126]	2013	40	41	100	NO	Triathlon running, >5 h/week for >2 yrs	0	
Bohm et al. [127]	2016	33	47	100	YES	Endurance, >10 h/week for > 10 yrs	3 (0 controls)	1: subepicardial strain
Tahir et al. [116]	2018	83	43	65	YES	Triathletes training ≥10 h/week, competitions in the previous 3 yrs	17 (only in males)	5: subepicardial 2: junctional
Tahir et al. [128]	2019	78	43	100	YES	Triathletes training ≥10 h/week, competitions in the previous 3 yrs	19 (3.5 controls)	7: subepicardial 6: mid-myocardial 2: junctional

### 3.6. Aortic Dilatation

As part of the physiological exercise adaptation, slight dilatation of the aorta has been observed, which is more expressed at Valsalva’s sinus level. However, according to high-volume studies, aortic dilatation exceeding 40 mm in men and 34 mm in women is rare, observed in only approximately 1% of young athletes [130,131]. Gati et al. performed a prospective study focusing on aortic root dilatation in healthy young (19 ± 5.9 years) athletes with a follow-up period of 5 ± 1.5 years. In their experience, athletes’ sinus of Valsalva was only 0.5 mm larger than the control subjects. Only 0.17% of male and 0.4% of female athletes showed an aortic diameter above 40 and 38 mm, respectively. During the follow-up period, none presented progressive enlargement of the aortic diameter compared to baseline [132]. In a study conducted in Italy on 2317 athletes, only 17 showed aortic root dilatation above 40 mm. During a follow-up period of 8 years, no aortic events occurred. However, in 3, the aorta dilated substantially (up to 50 mm) without the proven systemic disease [130].

Hence, aortic dilatation seems rare in athletes. However, there may be an increased risk for veteran athletes engaged in specific sports. Churchill et al. [133] studied 442 master rowers and runners (mean age 61 years, male 60%), and they observed a prevalence of 21% of aortic dilatation (31% men, 6% women), especially among rowers and elite competitors from both sports if compared with validated age, sex, and body-size-adjusted general population nomograms. According to the authors, long-term participation in competitive sport could be a risk for aortic dilatation of both sinuses of Valsalva and ascending aorta. Rowing is characterized by a combination of dynamic and static exercise, with repetitive surges in arterial blood pressure that can explain the higher prevalence of aortic dilatation in this subset of master-level athletes. Rugby players have been recently identified as another group at risk of aortopathy [134]. Kay et al. found an unexpectedly high prevalence (41%) of aortic root dilatation (>40 mm) in middle-aged (45 ± 13 years) former elite rugby players. They also found an association between aortic dilatation and a longer duration of competitive sports participation. In a multiple logistic regression model, a more extended history of competitive exercise (above 15 years) was associated with an aortic size of >40 mm, which involved adjusting for BSA and age. They emphasized the importance of vigilant screening in this group, as the clinical significance of these findings is currently unclear [135]. During heavy-resistance exercise, elite strength athletes are subject to a large increase in arterial blood pressure due to higher heart rate and cardiac output, and the Valsalva maneuver, which occurs during strength training. Bigi et al. compared this type of athlete with an age- and height-matched population, and they proved an increase in aortic root diameter, with impossible overlapping of aortic cusps leading to mild and moderate aortic regurgitation. Moreover, the high-intensity strength training duration correlated with aortic size [131,136].

In summary, participation in competitive sports does not seem to be associated with clinically relevant aortic dilatation, except for certain disciplines. However, in such athletes, the dilatation of the aorta may be a benign adaptation to physical exercise, and there is no evidence of an increased risk of progressive aortic aneurysm or aortic dissection.

### 3.7. Coronary Artery Calcifications

Exercise is the cornerstone of coronary artery disease (CAD) prevention. However, some evidence suggests that very prolonged and intense exercise may paradoxically favor coronary calcifications.

**Figure 6:**
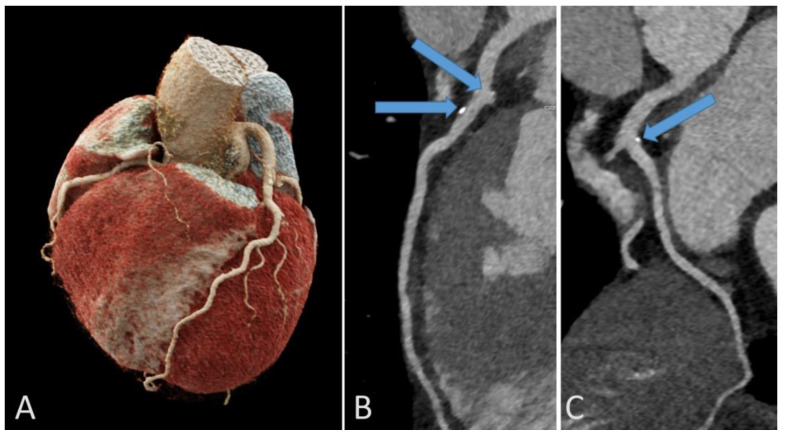
Representative example of coronary calcifications in a 56-year-old endurance athlete without risk factors who underwent coronary computed tomography for investigation of repolarization abnormalities on resting ECG. 3D reconstruction of the coronary artery tree (**A**). Calcific plaques in left anterior descending (arrows, **B**) and circumflex (arrow, **C**) coronary arteries.

A seminal study by Aengevaeren et al. [137] assessed coronary calcifications in middle-aged (55 ± 7 years) men with different lifetime training volumes. The 284 subjects were categorized according to their lifelong exercise volume in average MET-minutes/week. Coronary artery calcification was present in more than half (53%) of the athletes. Those with >2000 MET-min/week showed a significantly higher CAD burden than those with <1000 MET-min/week. The adjusted odds ratio for coronary artery calcification in this comparison was 3.2 in the >2000 MET-min group. Interestingly, the most active group presented more benign plaque compositions with a lower prevalence of mixed plaques (48% vs. 69%) and more often had only calcified plaques (38% vs. 16%) when compared with the least active group. Similar results were obtained by Merghani et al. [138], who enrolled 152 master endurance athletes (54.4 ± 8.5 years, 70% male) and 92 controls with low coronary risk. Even if most athletes showed no coronary calcifications, severe coronary calcifications (≥300 Agatston units) and coronary stenosis ≥50% showed a prevalence of 11.3% and 7.5%, respectively, significantly higher than non-athletes. In addition, 4.6% showed ischemic patterns of LGE on CMR. On the other hand, plaque characteristics differed between athletes, who demonstrated predominantly calcific plaques, and nonathletes, who showed predominantly mixed morphology plaques (Figure 6).

In summary, evidence is emerging that long-lasting endurance sports activity may promote coronary plaques in a subset of master male athletes compared with less active individuals with a similar coronary risk profile. However, such plaques are usually calcific and therefore at lower risk of rupture. Whether the increased prevalence of coronary stenosis in endurance athletes translated into a higher risk of acute coronary syndrome remains to be elucidated.

## 4. Conclusions

In conclusion, our review suggests that very intense sports activity may cause reversible electrocardiographic changes, myocardial dysfunction, and troponin elevation with complete recovery within a few days. The theory that repeated bouts of acute stress on the heart may lead to chronic myocardial damage creating a potentially dangerous arrhythmogenic substrate remains to be demonstrated. However, male, middle-aged individuals with a long, athletic career show an increased prevalence of cardiovascular abnormalities such as electrical conduction delay, AF, myocardial LGE, and coronary calcifications compared to non-athletes. However, the cause–effect relationship between such abnormalities and exercise and, most importantly, their prognostic relevance remains to be established. Moreover, evidence of any exercise-related adverse effects on the hearts of female athletes is lacking.

Pending future studies, we believe that when advising athletes about the pros and cons of exercise, we should apply the old Latin aphorism dosis sola facit venenum (“only the dose makes the poison”). There is no doubt that exercise is a medicine, and the recent European Society of Cardiology guidelines emphasized that adapted physical activity is beneficial for all cardiovascular patients, not just healthy individuals [88]. However, an increased number of middle-aged individuals desire to challenge their physical limits by engaging in ultra-endurance sports. We should warn these subjects that such extreme physical activities might damage not only their tendons and joints but also their hearts.

## Data Availability

Not applicable.
